# The Role of Health Technologies in Multicomponent Primary Care Interventions: Systematic Review

**DOI:** 10.2196/20195

**Published:** 2021-01-11

**Authors:** Geronimo Jimenez, David Matchar, Choon Huat Gerald Koh, Rianne van der Kleij, Niels H Chavannes, Josip Car

**Affiliations:** 1 Centre for Population Health Sciences (CePHaS) Lee Kong Chian School of Medicine Nanyang Technological University Singapore Singapore Singapore; 2 Department of Public Health and Primary Care Leiden University Medical Center Leiden Netherlands; 3 Programme in Health Services and Systems Research Duke-NUS Medical School Singapore Singapore; 4 Saw Swee Hock School of Public Health National University of Singapore Singapore Singapore

**Keywords:** digital health, health system improvements, health technologies, primary care, systematic review

## Abstract

**Background:**

Several countries around the world have implemented multicomponent interventions to enhance primary care, as a way of strengthening their health systems to cope with an aging chronically ill population and rising costs. Some of these efforts have included technology-based enhancements as one of the features to support the overall intervention, but their details and impacts have not been explored.

**Objective:**

This study aimed to identify the role of digital/health technologies within wider multifeature interventions that are aimed at enhancing primary care, and to describe their aims and stakeholders, types of technologies used, and potential impacts.

**Methods:**

A systematic review was performed following Cochrane guidelines. An electronic search, conducted on May 30, 2019, was supplemented with manual and grey literature searches in December 2019, to identify multicomponent interventions that included at least one technology-based enhancement. After title/abstract and full text screening, selected articles were assessed for quality based on their study design. A descriptive narrative synthesis was used for analysis and presentation of the results.

**Results:**

Of 37 articles, 14 (38%) described the inclusion of a technology-based innovation as part of their multicomponent interventions to enhance primary care. The most commonly identified technologies were the use of electronic health records, data monitoring technologies, and online portals with messaging platforms. The most common aim of these technologies was to improve continuity of care and comprehensiveness, which resulted in increased patient satisfaction, increased primary care visits compared to specialist visits, and the provision of more health prevention education and improved prescribing practices. Technologies seem also to increase costs and utilization for some parameters, such as increased consultation costs and increased number of drugs prescribed.

**Conclusions:**

Technologies and digital health have not played a major role within comprehensive innovation efforts aimed at enhancing primary care, reflecting that these technologies have not yet reached maturity or wider acceptance as a means for improving primary care. Stronger policy and financial support, and advocacy of key stakeholders are needed to encourage the introduction of efficient technological innovations, which are backed by evidence-based research, so that digital technologies can fulfill the promise of supporting strong sustainable primary care.

## Introduction

Primary care is often considered a cornerstone of health care systems. Health systems with strong primary health care produce better and more equitable health outcomes, are more efficient, and can achieve higher user satisfaction in comparison to health systems with only a weak primary care orientation [1,2]. Changing demographics, an increasingly aging population, and the increased burden of noncommunicable diseases have been identified as new challenges for health systems worldwide [3-5], and strengthening primary care has been proposed as one solution to address these challenges.

Many countries have implemented a wide array of innovations to enhance primary care, ranging from policy initiatives, such as capitated reimbursement, to ground level improvements, such as improving access to primary care practices and enhancing the roles of nurses to provide comprehensive primary care services [6-8]. As in other fields, such as finance, retail, and agriculture, an increasingly important domain for innovation involves the incorporation of technology. Technologies are having an impact on health service delivery and health system administration, and they promise to provide solutions for improving primary care [9,10].

There have been many studies emphasizing individual digital technologies for improving specific aspects of health care and primary care. Some of these include digital health assistants to help with administrative tasks, medical chatbots to engage patients more frequently, and the use of electronic health records and telemedicine, among others [9-11]. However, no studies have explored the role of technologies within multicomponent efforts to enhance primary care, that is, whether within initiatives comprised of several features aimed at enhancing primary care, there was a technology element being introduced, and if yes, what it was.

We aimed to systematically explore the role that health/digital technologies have played in multicomponent efforts designed to improve primary care by identifying (1) the types of technologies implemented, (2) the functional objective of the technology, (3) the relevant stakeholders, and (4) whether they have an impact on enhancing the defining features of primary care (ie, first contact, comprehensiveness, coordination, and continuity) [12], denoted here as the “4Cs.” We explored the overall outcomes of the multicomponent interventions in which technology is one component to attempt to discern the specific contribution of the technologies within these efforts. Textbox 1 provides useful definitions for concepts and terms that will be used throughout the article.

**Useful definitions.**

Multicomponent interventions/innovation environments: programs or strategies composed of several innovations/features to enhance primary care.

Innovation features: individual innovation elements included in multicomponent interventions.

Health technologies: application of scientific knowledge to solve health care–related problems, including its corresponding machinery and equipment (includes information technology, digital health, eHealth, mHealth, etc).

4Cs: the primary care core functions (first contact, comprehensiveness, coordination, and continuity).

Quadruple aim outcomes: the four types of outcomes to measure successful health system improvements (population health outcomes, health care utilization and cost outcomes, patient satisfaction, and provider satisfaction).

## Methods

A systematic review was designed and performed following Cochrane guidelines for conducting systematic reviews [13]. The detailed methods for this review are described in an article that explored multicomponent interventions aimed at enhancing primary care, which identified 18 innovation strategies and provided a broader picture of the many innovation features used internationally to improve various aspects of primary care simultaneously [14]. A summary is provided below.

An electronic database search was performed in order to identify (1) multicomponent interventions or “innovation environments” aimed at enhancing primary care (with at least three innovation features); (2) factors influencing at least one of the primary care core functions (4Cs), and (3) studies reporting on any of the four basic types of outcomes of a successful health system (the so-called “quadruple aim” outcomes of population health, health care costs and utilization, patient satisfaction, and provider satisfaction) [15] and providing numerical values for at least five outcome measures. In a previous scan of the literature, we identified many specific interventions aimed at enhancing a particular aspect of primary care services (eg, *the paper stamp checklist tool enhances asthma guideline knowledge and implementation by primary care physicians*), and based on this, we determined that consideration of studies describing interventions with at least three distinct innovation features and measuring at least five outcome measures could ensure that the interventions were indeed “multicomponent.”

A search strategy was developed, and it focused on the following three main sets of terms: (1) primary care–related terms; (2) innovation/reform/enhancement-related terms; and (3) study design filters (Multimedia Appendix 1). The electronic database search was performed in Ovid/MEDLINE on May 30, 2019, and it was supplemented by manual searches through the references of the included studies and by a grey literature search (ie, search through materials and documents produced by organizations outside of the traditional commercial or academic publishing and distribution channels, such as government and industry documents) in OpenGrey [16], using “primary care” and “innovation,” on December 12, 2019. From the studies fulfilling these criteria, we selected those that had technology-based enhancements as part of the elements in their multicomponent interventions.

We defined health technologies, using definitions from two World Health Organization reports, as the “application of scientific knowledge for practical purposes, including its corresponding machinery and equipment, to solve health care–related problems and improve quality of life” [17] and encompassing digital health technologies (the overarching term to include eHealth and mHealth, eg, telemedicine, electronic health records [EHRs], and wearable sensors) and their corresponding medical and assistive devices [9].

Quality evaluation of the included studies was based on study design, using the National Institutes of Health–National Health, Lung and Blood Institute’s “Study Quality Assessment Tools” [18], a comprehensive suite of study evaluation tools, which has been used in a variety of systematic reviews [19-21]. Data extraction was performed using a predefined data extraction form for study characteristics and general information (author/year, setting/country, policy influence, study design and quality, and patient population involved), primary care intervention elements, and quadruple aim outcomes, including reported magnitudes for each outcome measure. A narrative descriptive approach was utilized to identify and report the types and specific details of the implemented technologies, the involved stakeholders, whether and which 4Cs were arguably supported, and the outcomes influenced by the corresponding technology.

## Results

### Search Results

**Figure 1:**
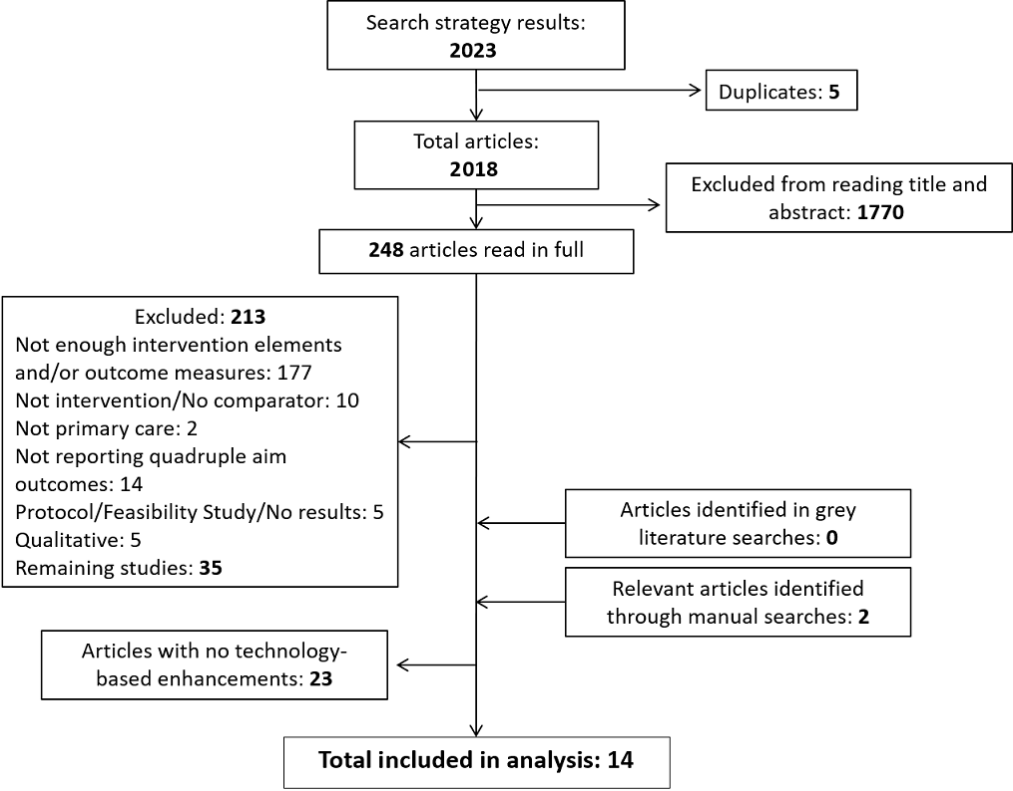
PRISMA diagram describing the study selection process. Figure extended from Jimenez et al [14].

After the electronic search, subjecting the articles to the inclusion/exclusion criteria and manual reference and grey literature searches resulted in 37 articles fulfilling the requirements for multicomponent interventions as described above. From these, 14 studies had technology-based enhancements and were included in the analysis (Figure 1).

### Study Characteristics

Articles were published between 2008 and 2017, and half of them were published since 2016. Most described studies performed in the United States (9/14, 64%). Additionally, four were from Europe (two from Germany and two from Spain) and one was from Argentina. Eight articles mentioned policies influencing the implementation of the innovation programs as broader country, regional, or organizational efforts to enhance primary care (Table 1) [22-35].

**Table 1:** Study characteristics organized by study type (N=14) (adapted from Jimenez et al [14]).

Study type	Author (Year)	Program name	Setting/context	Policy/ government program influencing innovation	Study design (quality evaluation rating)^a^	Patient population (if any)	Innovation elements included in the full intervention^b^	Types of outcomes studied
Controlled intervention study	Coderch et al (2016) [22]	—^c^	Integrated health care organization in the region of Girona, Spain in 2011 (128,000 residents)	Catalonia’s 2011-2015 health plan; creation of the Program for Chronic Condition Prevention and Care	Controlled, pragmatic, randomized clinical trial, with three arms: one blind control and two open intervention groups (fair)	Complex chronic patients who account for 5% of the highest risk of highest health costs each year	- Accountability mechanisms - Care plan development - Improved access - Improved specialty care access - Enhanced coordination/information exchange efforts - Provider education or training - Technology enhancements	HC^d^ costs and utilization
Controlled intervention study	Prestes et al (2017) [23]	DIAPREM study	Primary care units of La Matanza County, Argentina	—	Random selection of 30 PC^e^ providers and 30 nurses from 40 PC units (fair)	T2DM^f^ patients	- Efforts to improve performance monitoring - Enhanced continuity/transition-based efforts - Provider education or training - Technology enhancements	Population health HC costs and utilization
Controlled intervention study	Ruescas-Escolano et al (2014) [24]	PROPRESE Trial	Multicentric, PC study (15 health centers), participating in the Cardiometabolic Valencian Study	—	Open randomized clinical trial with 1-year follow-up (good)	Patients with ischemic heart disease	- Efforts to improve performance monitoring - Improved patient self-management - Provider education or training - Others - Team-based care - Technology enhancements	Population health
Observational cohort or cross-sectional study	Dale et al (2016) [25]	Comprehensive Primary Care (CPC) Initiative	A large and diverse set of practices in seven Center for Medicare and Medicaid Services (CMS) regions (four states and three metropolitan regions in the United States)	Launching of the Centers for Medicare and Medicaid Services’ Comprehensive Primary Care Initiative, in October 2012	Pre-post design with a comparison site (fair, classified as retrospective cohort for quality evaluation)	Medicare fee-for-service beneficiaries	- Care development plan - Case management - Improved access - Improved patient self-management - Payment-based enhancements - Social or community services engagement - Technology enhancements	HC costs and utilization Patient satisfaction
Observational cohort or cross-sectional study	Goff et al (2017) [26]	Buena Salud	Program implemented at Brightwood Health Center (BHC) in MA, an urban community health center with a largely Hispanic population (88%) insured primarily by either Medicaid (59%) or Medicare (28%)	—	Controlled before-and-after study (fair)	T2DM patients enrolled in the Buena Salud program	- Accountability mechanisms - Case management - Improved access - Improved patient self-management - Improved specialty care access - Social or community services engagement - Team-based care - Technology enhancements	Population health
Observational cohort or cross-sectional study	Maeng et al (2013) [27]	ProvenHealth Navigator	36 Geisinger-owned PC practices, as well as seven contracted PC practices in GHP’s^g^ provider network. Geisinger’s regional health care system is a provider to central, south-central, and northeastern Pennsylvania and southern New Jersey	PCMH^h^ transformation in primary care	Survey of patients in “PHN^i^ sites.” A comparable survey of patients from non-PHN sites was conducted for comparison (fair)	General patient population of PC practices enrolled in the PHN program	- Case management - Efforts to improve performance monitoring - Enhanced service capacity - Improved access - Improved patient self-management - Payment-based enhancements - Social or community services engagement - Team-based care - Technology enhancements	Patient satisfaction
Observational cohort or cross-sectional study	Maeng et al (2012) [28]	ProvenHealth Navigator	36 Geisinger-owned PC practices, as well as seven contracted PC practices in GHP’s provider network. Geisinger’s regional health care system is a provider to central, south-central, and northeastern Pennsylvania and southern New Jersey	PCMH transformation in primary care	Multivariate logistic regression models with controls (members not in the program) (fair, classified as retrospective cohort for quality evaluation)	General patient population of PC practices enrolled in the PHN program	- Case management - Efforts to improve performance monitoring - Enhanced service capacity - Improved access - Improved patient self-management - Payment-based enhancements - Social or community services engagement - Team-based care - Technology enhancements	Population health
Observational cohort or cross-sectional study	Phillips et al (2014) [29]	The Illinois Medicaid Health Connect and Your Healthcare Plus programs	Illinois Medicaid beneficiaries, corresponding to 15% of the total state population	The Memisovski v. Maram suit (2004) ruled that Illinois had violated federal law by not providing adequate access to PC services for its Medicaid population, which made Illinois an early leader in Medicaid reform	Analysis of Medicaid claims and enrollment data from 2004 to 2010, covering both pre- and post-implementation (good, classified as retrospective cohort for quality evaluation)	Medicaid beneficiaries	- Accountability mechanisms - Care plan development - Care management - Improved access - Payment-based enhancements - Provider education or training - Technology enhancements	HC costs and utilization
Observational cohort or cross-sectional study	Wensing et al (2017) [30]	GP^j^-centered care (GPCC) program	Introduction of a program to enhance the role of general practice for patients with chronic diseases in Baden-Wuerttemberg, a German federal state with about 10.7 million inhabitants.	—	Comparative evaluation based on two cross-sectional studies at 4 and 5 years after its start (T1 and T2, respectively), based on data continuously collected for administrative control and reimbursement purposes (good)	General population aged 18 years or older with at least one primary care visit	- Accountability mechanisms - Efforts to improve performance monitoring - Enhanced coordination/ information exchange efforts - Improved access - Improved patient self-management - Inclusion of new/enhanced roles - Payment-based enhancements - Pharmacy/medication-related efforts - Provider education or training - Team-based care - Technology enhancements	HC costs and utilization
Case-control study	Freytag et al (2016) [31]	GP-centered program	A major Statutory Health Insurance fund AOK PLUS^k^, which covers 41% of the population in central Germany, established a GP-centered health care program in 2011 in the German federal state of Thuringia	In Germany, enhanced primary care programs started in 2004 with the creation of a legal framework to support “GP-centered health care”	Retrospective case-control study based on insurance claims data (fair)	General patient population	- Inclusion of new/enhanced roles - Payment-based enhancements - Pharmacy/medication-related efforts - Provider education or training - Technology enhancements	HC costs and utilization
Pre-post study with no control	Conrad et al (2016) [32]	Group Health Cooperative’s Access Initiative	PC practices within the integrated care delivery system that serves the Puget Sound region in Washington state	—	Pre-post implementation productivity assessment (good)	Group health cooperative’s enrollees	- Enhanced service capacity - Improved access - Improved specialty care access - Others - Payment-based enhancements - Technology enhancements	HC costs and utilization
Pre-post study with no control	Engel et al (2016) [33]	Geriatrics in Primary Care (GPC)	Two large medical center practices at the Veterans Affairs Boston Healthcare System in 2014	Adoption of the Patient Aligned Care Team model of care, which is adapted from the PCMH, by the Veterans Affairs	Before-after evaluation of chart reviews (poor)	Veterans from the Veterans Affairs health system Boston, enrolled in the program	- Case/care management - Enhanced continuity/transition-based efforts - Enhanced service capacity - Improved access - Team-based care - Technology enhancements	HC costs and utilization
Pre-post study with no control	Maeng et al (2012) [34]	ProvenHealth Navigator	36 Geisinger-owned PC practices, and seven contracted PC practices in GHP’s provider network. Geisinger’s regional health care system is a provider to regions of Pennsylvania and New Jersey	PCMH transformation in primary care.	Pre-post (measured at six points) and member fixed-effects model to measure within-member variation in the total cost and the PHN exposure variable over time (good)	GHP’s Medicare Advantage plan members who were at least 65 years and enrolled in clinics that became PHN sites	- Case management - Efforts to improve performance monitoring - Enhanced service capacity - Improved access - Improved patient self-management - Payment-based enhancements - Social or community services engagement - Team-based care - Technology enhancements	HC costs and utilization
Pre-post study with no control	Ralston et al (2009) [35]	Group Health’s Access Initiative	Adult respondents (aged ≥18 years) receiving care in Group Health’s Western Washington Integrated Delivery System	Patient-centered system reforms (such as the PCMH model of 2007) mentioned as a shift in the way access to PC is provided, which encouraged HMOs^l^ to change their restrictive access system.	Program impact evaluation, evaluating at three time points, based on the implementation dates of the initiative’s components (fair)	Adult respondents (aged ≥18 years) receiving care in Group Health’s Western Washington Integrated Delivery System	- Accountability mechanisms - Improved access - Improved specialty care access - Others - Payment-based enhancements - Technology enhancements	HC costs and utilization Patient satisfaction Provider satisfaction

^a^Ratings: good/fair/poor. Study type linked to the tool used for quality evaluation.

^b^Full details of innovation elements are provided in Multimedia Appendix 2.

^c^Not available or not reported in the articles.

^d^HC: health care.

^e^PC: primary care.

^f^T2DM: type 2 diabetes mellitus.

^g^GHP: Geisinger Health Plan.

^h^PCMH: Patient-Centered Medical Home.

^i^PHN: PatienHealthNavigator.

^j^GP: general practitioner.

^k^AOK PLUS: health insurance scheme under Germany insurer AOK.

^l^HMO: health maintenance organization.

In terms of study designs and quality evaluation results, three publications reported controlled interventions (two of “fair” and one of “good” quality), six reported observational cohort or cross-sectional studies with controls (four of “fair” and two of “good” quality), one reported a case-control study of “fair” quality, and four reported pre-post studies without controls (one of “poor”, one of “fair,” and two of “good” quality). Populations studied or linked to the results included the general population enrolled in the programs (in six articles), chronically ill patients with one disease or complex chronic patients (in four articles), and special populations, including elderly and disadvantaged populations (in four articles).

The interventions in the articles included between four and 11 “innovation features” (see Multimedia Appendix 2 for definitions). The average number of features per intervention was seven (median seven), and the most common types, beside technology-based enhancements (present in all interventions), were innovations to improve access (in 11 articles), payment-based enhancements (in nine articles), and care/case management (in seven articles). In terms of the types of outcomes, the most commonly reported was health care costs and utilization (in 10 articles), followed by population health outcomes (in four articles), patient satisfaction (in three articles), and provider satisfaction (in one article). These are not mutually exclusive as one article reported on three outcomes and two reported on two outcomes each. The remaining 11 articles reported on one outcome each.

### Technology-Based Results

Of the 37 articles, 14 (38%) describing multicomponent interventions to enhance primary care included technology-based enhancements as one of the innovation elements (Table 2) [22-35].

**Table 2:** Technology types and details, aims, stakeholders involved, 4C support, and outcome summary (N=14).

Study	Technology-based on	Specific technology innovation	Aim and stakeholder (patient/provider/admin manager)	“4C” being supported by technology	General results and direction of the effects on quadruple health outcomes (of the full intervention)^a^
Coderch et al (2016) [22]	EMRs^b^	- Identification of patients: complex chronic patients are identified by labelling them in unique EMRs for providers - Proactive actions in PC^c^: individualized care plan registered in unique EMRs for providers	For providers, to be able to easily identify complex chronic patients under their care	Continuity	Health care costs and utilization ↑ (considerable increase in nonurgent primary care visits for partial and full interventions compared to each other and to control for both years 1 and 2) ↔ (mixed results for acute hospital admission and stay for year 1: considerable decrease for partial intervention compared to control, but considerable increase in full intervention compared to partial intervention; similar for readmissions <30 days in year 2, and considerable decrease for partial intervention and increase for full intervention when compared to each other) ↓ (increase in the number of prescriptions for full intervention compared to control for year 2)
Conrad et al (2008) [32]	Online messaging platform Online patient portal/website	- Patient-provider secure messaging through the MyGroupHealth enrollee website, including physician financial incentives for secure messaging patients - Internet access for enrollees to their EMRs through MyGroupHealth - Health promotion information on the MyGroupHealth secure website	For providers and patients, to have enhanced communication For patients, to promote self-management (through access to their medical information and health promotion information)	Continuity Comprehensiveness	Health care costs and utilization ↑ (considerable increase in panel size per FTE^d^ and relative value unit per visit; considerable decrease in visits per FTE and per member per quarter costs) ↔ (nonrelevant increase in relative value unit per FTE)
Dale et al (2016) [25]	EMRs	- Optimal use of health IT^e^, including improving EHR^f^ function and capability and developing practice capability for optimal use of EHR; enabling exchange of patient information to support care; and developing quality measurement and reporting from EHRs	For providers, to better use EHRs, use information to support patient care, and improve quality monitoring	Comprehensiveness Coordination Continuity	Health care costs and utilization ↑ (decrease in total Medicare expenditures [without initiative care-management fees] and considerable decrease in PC visits and diabetes patients with no tests performed) ↔ (nonrelevant effects for hospitalizations, ED^g^ visits, specialist visits, admissions for ambulatory care–sensitive conditions, and likelihood of readmissions; no differences for tests performed for diabetes or ischemic vascular patients) Patient satisfaction ↑ (increase in satisfaction with timely appointments, self-management support, and discussion of medications) ↔ (nonrelevant differences for communication with providers, knowledge of providers of other services, and patient ratings of providers)
Engel et al (2016) [33]	Telephone Electronic consultations	- Proactive telephone contact with veterans and caregivers, ready access to primary care colleagues, and informed use of telephone follow-up to enhance care while reducing nonessential clinic visits - Electronic consultation for formal referrals to geriatrics in PC program	For providers, to have easier referral to services For patients, to reduce clinical visits, while enhancing care	First contact Continuity Coordination	Health care costs and utilization ↑ (decrease in the number of specialist visits after years 1 and 2, while maintaining the number of PC visits)
Freytag et al (2016) [31]	Medication-specific IT tool	- Obligatory use of a specific IT-pharmacotherapy tool to support rational pharmacotherapy	For providers, to support rational prescription of medicines	Comprehensiveness	Health care costs and utilization ↑ (decrease in the cost of drug prescriptions; increase in GP^h^ consultations and decrease in specialist consultations, hospital use, and remedies; decrease in share of patients consulting more than one GP and accessing specialist without referrals; increase in the number of patients in disease management program and home visits; and decrease in the number of medical check-ups) ↓ (increase in the cost of GP consultations and specialist consultations and increase in the share of patients with five or more different medications) ↔ (no change in the number of ED hospitalizations or increase in the nursery care level)
Goff et al (2017) [26]	EHRs Use of insurer data	- Use of electronic health registries to identify patients in need of care and services (quarterly, reviewed the data contained in EHRs and insurer data focusing on specific care parameters in care [ie, ordered labs and mammography, scheduled PC visits, etc])	For providers, to monitor care needs and ensure tests and visits	Continuity	Population health ↑ (considerable changes in the mean DBP^i^ and microalbumin/creatinine ratio test within 12 months) ↔ (no relevant difference for changes in HbA_1c_ measures, lipid measures, or other blood pressure measures; changes for HbA_1c_ tests and lipid panels)
Maeng et al (2012, 2012, 2013) [27,28,34]	EHRs Online patient portal Online messaging platform Modeling and utilization data tools	- Preventive and chronic care optimized by health IT. - Active delivery of information to other team members at the point of care via shared EHRs - Access to the patient portal for reviewing medical records and secure messaging with providers - Predictive modelling and utilization of data tools and normative management data to improve care	For providers, to have availability of patient information for all medical team members For providers and patients, to have enhanced communication For patients, to have access to their medical records to promote self-management For practices, to have improved monitoring for population care	Comprehensiveness Coordination Continuity	Population health ↑ (decrease in amputation and end-stage renal disease in the intervention group) ↔ (no difference for myocardial infarction or stroke) Health care costs and utilization ↑ (decrease in the per member and per month allowed costs; considerable overall savings with and without Rx coverage interaction) ↓ (increase in the cost of Rx coverage, without considering other program costs) Patient satisfaction ↑ (improvement in perceived changes in care delivery, ie, “noticed difference in care coordination and higher quality,” increase in reporting of doctor’s office as usual care, and decrease in ER^j^ visits) ↔ (no relevant changes for access to care or primary care provider performance)
Phillips et al (2014) [29]	Online registries/report cards	- Multiple online tools, such as registries and report cards, to assist clinicians with population-based management	For providers, to have improved monitoring and population-based management	Continuity	Health care costs and utilization ↑ (increase in estimated cost savings and rate estimated annual savings; decrease in hospitalization, bed-day, and avoidable hospitalization rates; and increase in all quality measure changes [test and screenings]) ↔ (decrease in the ED visit rate for IHC^k^ but increase for YHP^l^)
Prestes et al (2017) [23]	Data monitoring system	- The QUALIDIAB data system was used to verify the impact of the diabetes education intervention, and the data collected are useful to allocate resources (human and financial) considering real demand	For providers, to verify the impact of the intervention and allocate resources using collected data	Continuity	Population health ↑ (considerable improvements for DBP, glycemia, HbA_1c_, total cholesterol, and LDL-c^m^ and increase in the percentage of patients with target SBP^n^ and HbA_1c_ levels) ↔ (nonrelevant differences for SBP, creatinine, proteinuria, HDL-c^o^, DBP <80 mmHg, glycemia <100 mg/dL, cholesterol <200 mg/dL, and triglyceride <150 mg/dL) Health care costs and utilization ↑ (considerable increase in dyslipidemia patients treated, eye tests, and cardiovascular evaluations) ↔ (nonrelevant differences for dyslipidemia treated under target or any hypertension treatments)
Ralston et al (2009) [35]	Online patient portal Online messaging platform	- Web access for patients that provides secure email with physicians, medical record access, medication refills, appointment scheduling, discussion groups, and health promotion information	For patients, to facilitate accessing physicians, making appointments, refilling prescriptions, accessing medical records, and supporting self-management	First contact Comprehensiveness	Health care costs and utilization ↑ (improvement in “Getting Needed Care” and “Getting Care Quickly” scores) Patient satisfaction ↑ (improvement in satisfaction with the ability to see a personal doctor; time spent on the phone and waiting time for appointment; ease of getting care; and ratings of health care, health plan, and opinion of Group Health) Provider satisfaction ↑ (improvement in the perception of providers toward Group Health’s quality and services provided and for Group Health as a good place to work)
Ruescas-Escolano et al (2014) [24]	EMRs	- Use of unique EMRs that allow for following control indicators and risk stratification	For providers, to monitor patient progress and manage risk	Continuity	Population health ↑ (considerable improvements in smoking status, cholesterol, and SBP) ↔ (nonrelevant differences for DBP)
Wensing et al (2017) [30]	Medication-specific IT tool Updated IT systems	- The practice has a data-orientated quality system and decision support for prescribing medication; prompts in software to support use of generic and discounted drugs - The practice has up-to-date IT	For providers, to support medication prescription and promote generic medication use For practices, to have better organization to support easier patient access	Comprehensiveness First contact	Health care costs and utilization ↑ (decrease in the costs of medication therapy and hospital admissions) ↑ (increase in the number of visits to family physicians and mean number of prescription drugs; decrease in the number of prescriptions that should be avoided, contacts with specialists with and without referrals, hospital admissions, avoidable hospital admissions, number of days at hospital, and readmissions)

^a^Extracted from Jimenez et al [14].

^b^EMR: electronic medical record.

^c^PC: primary care.

^d^FTE: full-time equivalent.

^e^IT: information technology.

^f^EHR: electronic health record.

^g^ED: emergency department.

^h^GP: general practitioner.

^i^DBP: diastolic blood pressure.

^j^ER: emergency room.

^k^IHC: Illinois Medicaid Health Connect.

^l^YHP: Your Healthcare Plus.

^m^LDL-c: low-density lipoprotein cholesterol.

^n^SBP: systolic blood pressure.

^o^HDL-c: high-density lipoprotein cholesterol.

According to the descriptions of the articles, we were able to identify the following six broad categories for the types of implemented technologies (description below includes intended stakeholder and use):

1. Enhancements leveraging *electronic medical/health records* [22,24-28,34]: it was the most common category (reported in seven studies from five interventions) and was aimed at providers. Their use is related to identifying specific groups of patients (eg, chronically ill) or specific needs of patients (eg, services needed), exchanging patient information, and developing quality measurements/control and risk stratification.
2. *Data monitoring technologies/online registries* [23,26-29,34]: it was the second most common category (in six studies from four interventions) and was aimed at providers and practices. It was related to the management of utilization data to allocate resources and improve care, help with population-based management, and check on the impact of programs.
3. Web-based *online portals and messaging platforms* [27,28,32,34,35]: it was included in five studies (reporting on three interventions) and was aimed at patients to access their medical records, obtain additional health promotion information, promote self-management, and facilitate access and communication with providers.
4. *Medication-specific eHealth/information technology tools* [30,31]: it was included in two studies and was aimed at providers to support pharmacotherapy and medication prescription.
5. *Telephone-based enhancements* [33]: it was described in one article and was aimed at providers to communicate with patients and caregivers, and provide follow-up to reduce patients’ nonessential clinic visits.
6. *Electronic consultations* between providers [33]: it was described in one study and was aimed at enhancing geriatric referrals.

Based on the description of the technological enhancements included in the studies, we were able to link them to the 4Cs in the following way:

1. *First contact*: Three programs aimed to apply technology to impact this feature through telephone-facilitated access to primary care colleagues, facilitated appointment scheduling through web portals, and updated digital health systems for easier patient access.
2. *Comprehensiveness*: Six interventions sought to increase the ability to manage a wider range of problems with technology, including providing additional health promotion information through patients’ web portals and enhancing capacity for providers to better use electronic medical records, improve medication prescription, and provide improved preventive and chronic care.
3. *Coordination*: Three programs used technology to improve care coordination by improving EHR-enabled information exchange and by allowing electronic consultations to facilitate care among primary care providers and specialists.
4. *Continuity*: Nine interventions sought to enhance the longitudinal relationship between patients and providers by enhancing the identification and follow-up of patients for individualized care, allowing more comprehensive identification and monitoring of service needs, and improving communication between patients and providers via online messaging or telephone contact.

### Outcomes

Since these technology-based innovation elements are part of wider innovation environments, which include additional enhancement features, it was not possible to attribute outcomes specifically to the identified technologies. However, we still present the outcomes of the full innovation environments in an effort to elucidate the potential role of these technologies in the outcomes. The numerical magnitudes for each outcome are presented in Multimedia Appendix 3 (along with details of the full intervention). Table 2 and the paragraphs below present a descriptive summary and general direction of the effects for these outcomes.

Overall, the studies presented mixed results (ie, nonsignificant changes or significant benefits and deteriorations simultaneously for a specific outcome) for all types of outcomes, except for provider satisfaction, which was reported only in one study. The most consistent improvements per type of outcome were as follows: (1) *health care costs and utilization*, increased cost saving and decreased costs for some parameters (eg, Medicare expenditure decreased by US $11 per beneficiary per month [25] and drug prescriptions decreased by €44 per patient [31]) and increased primary care visits compared to specialists; (2) *population health*, improved blood pressure control, improved glycated hemoglobin, decreased amputations and end-stage renal disease, and decreased smoking status; (3) *patient satisfaction*, increased satisfaction with timely appointments and self-management support and increased satisfaction with the ability to see the usual doctor; (4) *provider satisfaction*, improved perception toward place of work’s quality and services provided, and its consideration as a good place to work.

The most consistent mixed results by the type of outcome were as follows: (1) *health care costs and utilization*, nonsignificant changes or simultaneous improvements and deteriorations depending on the study for hospital admissions, readmissions, and emergency department visits; (2) *population health outcomes*, nonsignificant changes for cholesterol and lipid levels, myocardial infarction, and stroke; (3) *patient satisfaction*, no differences for communication with providers and for primary care provider performance.

The most consistent deteriorations were found for some health care costs and utilization outcomes, such as increased number of prescriptions, increased costs for general practitioner (eg, intervention €27 more expensive than control per patient) and specialist (intervention €22 more expensive than control per patient) consultations [31], and increased costs for prescription coverage.

## Discussion

### Principal Findings

Only 38% of our identified multicomponent interventions that aimed at enhancing primary care included technology-based enhancements, highlighting the fact that technology has not played a major role in comprehensive efforts aimed at enhancing primary care. This is not surprising, as it has been widely acknowledged that innovation in health care has always been difficult [36], especially if it has involved digital or technological efforts [37-39].

Most of the included articles reported on health care costs and utilization outcomes, signaling that technology-based efforts are either aimed at decreasing costs and utilization or at least not increasing costs without contributing to other aspects of system success. In fact, the only considerable unintended consequences were increased costs for general practitioner and specialist visits, and increased costs of prescription coverage (in some studies), suggesting that introducing technologies in health care can lead to increased costs, as it has been consistently reported in the literature [40-42].

The most common technology identified within these efforts was EHR, which is also not surprising given the widespread advocacy for this technology [43,44], and it was aimed mainly at providers or practices to facilitate information exchange among them and improve monitoring efforts. The only identified technology aimed at patients was the deployment of online patient portals, where they can see their records, message their providers, and access additional health information mostly for health promotion, which is in line with the idea that patients are ever more active participants in their own health care [44,45].

When analyzing the interventions in terms of their impact on the 4Cs, the technologies implemented were mostly aimed at improving continuity by increasing the identification and follow-up of patients (with labels in EHRs and telephone communication), enhancing monitoring efforts for identifying care and service needs (also mostly through EHRs and online registries), and ensuring more constant communication between providers and patients via online messaging. This reflects the growing importance of continuity of care, which in the past has had weak evidence linked to its benefits, but was recently highlighted as important, especially with regard to its link to decreased mortality risk [46,47]. Technologies have been promoted to improve comprehensiveness by providing additional health promotion information for patients, improving the ability of providers to prescribe medications, reinforcing the ability of primary care providers to cover a broader number of issues themselves, and avoiding overreferring [47].

In terms of outcomes, the literature provides limited but useful information. For example, increased patient satisfaction with timeliness of care, scheduling, and better self-management support could be in part explained by the use of online patient portals. Such portals allow patients to schedule appointments, see their own medical records, and access additional prevention information. Increased primary care visits, relative to specialist visits, appear to result from innovations that enhance monitoring of services needed and follow-up of patients (identified through EHRs and/or by telephone follow-up). The introduction of medication-specific digital/information technology tools could be associated with differing impacts. While studies reported a decrease in the costs of drug prescriptions and medication therapies, they also reported an increase in the mean number of drugs prescribed, and it was also associated with more costly consultations (around €25 extra per consultation [31]).

In order for digital technologies to play a more prominent role in primary care enhancement efforts, there is first a need for a responsible policy to support their development and introduction [48]. For example, some of the primary care enhancement environments have included an explicit policy encouraging the introduction of technology or information technology initiatives as part of their efforts [25,31,49]. To make this happen successfully, the technology must be seen as a tool that provides needed functions in a way that is effective, humane, and sustainable. Here, the context in which the technology will be implemented must be considered. It is essential to engage relevant stakeholders to deeply understand their environment and capabilities so that the introduced technology will be truly useful, improve (or at least not disrupt) existing workflow, and have tangible value [37,50]. In order to establish value, there is a need for technologies to be linked to evidence-based positive outcomes, taking into account their potential to improve health outcomes, costs, and patient and provider satisfaction.

There are some limitations for this study. The nature of the search and the specific requirements for including studies (ie, those describing multicomponent interventions aimed at enhancing primary care, which provided numerical magnitudes for reporting quadruple aim outcomes) may have made us overlook other important technological innovations aimed at improving primary care that had qualitative assessments only or did not measure quadruple aim outcomes. Similarly, since this review only focused on published and grey literature, it did not account for quality improvement interventions implemented at, for example, private primary care or accountable care organizations, which may have included technological innovations but no published results. Therefore, although technology may have a more prominent role in primary care–enhancing initiatives overall, the results from published and grey literature do not indicate so. Additionally, the fact that technological enhancements were one of many components within a primary care enhancement effort, our study eligibility criteria did not allow us to establish the actual and specific impact of the technologies on outcomes. However, it did help to situate these technologies within multicomponent innovation strategies and to gain preliminary insights into how technological enhancements may support other nontechnologically based innovation features and their impacts on the four primary care functions.

Taking all of this into account, future research should try to pinpoint the specific impact of technology-based innovation features within wider efforts aimed at enhancing primary care. This would mean including specific measures that could link outcomes to the use of such technology and quantify this impact. This exercise would also help to identify which of the 4Cs of primary care is being impacted by this technology, which would help understand the mechanisms of how these innovations are improving care. An additionally interesting research direction would be to explore how technological innovations are being leveraged at primary care private practices and/or accountable care organizations, to understand the actual role of technology in quality improvement initiatives for which there is no publicly available data. Such research could provide a more balanced view of the actual usage of technological innovations in primary care at the ground level.

### Conclusions

Although technology and digital health have been proposed and encouraged as possible solutions to improve primary care, they have not played a major role in formally evaluated multicomponent interventions aimed at enhancing primary care, as reflected in the published and grey literature. Other types of nontechnologically based innovations, such as those aimed at improving access, restructuring payments for providers, and providing team-based care, have been much more widely implemented, reflecting that digital health technologies have not yet reached maturity or wider acceptance as a means for improving primary care. Leveraging technologies already in use, such as EHRs, and internet-based technologies, such as online patient portals, seems to provide promising avenues to improve continuity and comprehensiveness in primary care, which may eventually lead to better health outcomes and improved patient satisfaction. A stronger push is needed if technologies are meant to support wider efforts aimed at enhancing primary care and for them to play a more substantial role within these efforts. High-level policy and financial support must be designed to focus on the needs of a diversity of stakeholders and to encourage evidence-based research based on a coherent set of methods and measures. In this way, we can hope to fulfill the promise of technologies and digital health to enhance health care through strong sustainable primary care.

## Appendix

Multimedia Appendix 1 Search strategy.

Multimedia Appendix 2 Innovation elements and definitions.

Multimedia Appendix 3 Details of interventions and magnitudes of outcomes.

## Abbreviations

EHR: electronic health record
