# Supplementary material for: The Role of Health Technologies in Multicomponent Primary Care Interventions: Systematic Review
Source: J Med Internet Res. 2021 Jan 11;23(1):e20195. doi: 10.2196/20195 (PMC7834942; doi:10.2196/20195)
Supplement: Multimedia Appendix 1 [file jmir_v23i1e20195_app1.docx]

**Multimedia Appendix 1: Search strategy and results**

Database: Ovid MEDLINE(R) and Epub Ahead of Print, In-Process & Other Non-Indexed Citations, Daily and Versions(R) <1946 to May 29, 2019>

Performed: 30/05/2019

--------------------------------------------------------------------------------

1 Primary health care/ (71981)

2 Physicians, Family/ (16020)

3 exp General Practice/ (73214)

4 ((community or family or general or group) adj2 (doctor? or physician? or practice? or practitioner?)).ti,ab. (126628)

5 "primary care".ti,ab. (104295)

6 1 or 2 or 3 or 4 (222818)

7 5 and 6 (56673)

8 enhance*.ti,ab. (1258706)

9 (new and (care adj1 model*)).ti,ab. (1297)

10 (reform* adj (health or service* or care or healthcare)).ti,ab. (527)

11 (service* adj1 redesign*).ti,ab. (255)

12 ((health or healthcare or service*) adj reform*).ti. (3113)

13 *Health Care Reform/ (22248)

14 *Organizational Innovation/ (4861)

15 *Quality Improvement/ (10807)

16 *Accountable Care Organizations/ (1018)

17 *Delivery of Health Care, Integrated/ (8608)

18 8 or 9 or 10 or 11 or 12 or 13 or 14 or 15 or 16 or 17 (1306310)

19 7 and 18 (4401)

20 evaluation studies/ or evaluation studies as topic/ or program evaluation/ or ((pre- adj5 post-) or (pretest adj5 posttest) or (program* adj6 evaluat*)).ti,ab. or (effectiveness or intervention).ti,ab. (1351302)

21 ("comparative study" or "evaluation studies").pt. or evaluation studies as topic/ or Pilot projects/ or program evaluation/ or Intervention Studies/ or ((pre- adj5 post-) or (pretest adj5 posttest) or (program* adj6 evaluat*)).ti,ab. or (effectiveness or intervention).ti,ab. (3081694)

22 ("clinical trial" or "clinical trial, phase i" or "clinical trial, phase ii" or clinical trial, phase iii or clinical trial, phase iv or controlled clinical trial or "multicenter study" or "randomized controlled trial").pt. or double-blind method/ or clinical trials as topic/ or clinical trials, phase i as topic/ or clinical trials, phase ii as topic/ or clinical trials, phase iii as topic/ or clinical trials, phase iv as topic/ or controlled clinical trials as topic/ or randomized controlled trials as topic/ or early termination of clinical trials as topic/ or multicenter studies as topic/ or ((randomi?ed adj7 trial*) or (controlled adj3 trial*) or (clinical adj2 trial*) or ((single or doubl* or tripl* or treb*) and (blind* or mask*))).ti,ab,kw. or ("4 arm" or "four arm").ti,ab,kw. (1534380)

23 observational study.mp. [mp=title, abstract, original title, name of substance word, subject heading word, floating sub-heading word, keyword heading word, organism supplementary concept word, protocol supplementary concept word, rare disease supplementary concept word, unique identifier, synonyms] (109825)

24 Case-Control Studies/ or Control Groups/ or Matched-Pair Analysis/ or retrospective studies/ or ((case* adj5 control*) or (case adj3 comparison*) or control group*).ti,ab,kw. (1437502)

25 20 or 21 or 22 or 23 or 24 (5135621)

26 19 and 25 (2023)
