# Supplementary material for: The Role of Health Technologies in Multicomponent Primary Care Interventions: Systematic Review
Source: J Med Internet Res. 2021 Jan 11;23(1):e20195. doi: 10.2196/20195 (PMC7834942; doi:10.2196/20195)
Supplement: Multimedia Appendix 2 [file jmir_v23i1e20195_app2.docx]

Multimedia Appendix 2: Innovation elements and definitions

| **Category** | **Definitions** |
| --- | --- |
| Accountability mechanisms | Programs/systems to identify a population for which a PC provider/practice was responsible for (e.g. empanelment, registries, incentives to enroll patients, etc.). |
| Care plan development | Creation of plans for patient care. |
| Case/care management | Innovations that include case management fees or include the addition of a case manager (e.g. risk-stratified case management). |
| Efforts to improve performance monitoring/ appraisal | Programs/systems that added or changed quality measures, or the way these were measured and identified. |
| Enhanced continuity/ transition-based efforts | Programs/systems designed to follow-up with patients or support in transitioning through different care levels (e.g. routine monitoring to identify changes in patient’s conditions; transition coaches, etc.). |
| Enhanced coordination/ information exchange efforts | Systems designed to improve the coordination and information exchange between different levels of care (e.g. care coordination fees, enhanced referral systems, etc.). |
| Enhanced service capacity | Innovations aimed at expanding the services provided at a PC site (e.g. equip PC clinic to handle emergencies, add geriatric services, add preventive care services, etc.). |
| Improved access | Systems facilitating access to PC services (i.e. expansion of service hours, telephone/web access, home-visits, etc.). |
| Improved patient self-management/ engagement | Programs/innovations aimed at engaging patients/caregivers in their own care (e.g. education or coaching, shared decision-making, etc.). |
| Improved specialty care access/support | Innovation aimed at facilitating access to specialists (e.g. removal of PC gatekeeping, adding specialists to PC clinic, etc.). |
| Inclusion of new/ enhanced roles | Adding new roles to the PC practices (e.g. healthcare assistants, practice facilitators, etc.) or enhancing existing roles (e.g. nurse acting as care manager). |
| Increased control of workload | Enhancements aimed at alleviating physicians caseloads by shifting activities to other team members. |
| Payment-based enhancements | Innovations related to changing the way providers get paid, including monetary incentives and compensation formulas, etc. (e.g. fee-for-service vs. capitation vs. pay-for-performance, etc.). |
| Pharmacy/medication-related efforts | Programs related to improving pharmacy or medication prescription, use of IT-pharmacotherapy tools, efforts to avoid duplicate medications, etc. |
| Provider education or training | Programs aimed to improve PC services by educating or training PC health professionals. |
| Social or community services engagement | Systems aimed at engaging community-based or social services. |
| Team-based care | Systems where care is provided by a team of providers. |
| Technology enhancements | Innovations where a technology was introduced to improve services (e.g. shared EMR across different providers, IT system for data driven improvements, online tools for a variety of enhanced capabilities, etc.). |
| Others | Innovations not classified in other categories and include: alternative medicine initiatives, enhanced screenings, redesign of service/organizational interventions to reduce variation in physician productivity, etc. |
