# Supplementary material for: The Role of Health Technologies in Multicomponent Primary Care Interventions: Systematic Review
Source: J Med Internet Res. 2021 Jan 11;23(1):e20195. doi: 10.2196/20195 (PMC7834942; doi:10.2196/20195)
Supplement: Multimedia Appendix 3 [file jmir_v23i1e20195_app3.docx]

**Multimedia Appendix 3: Primary care innovations’ details and magnitude of outcomes reported, by study design**

| Author (year) | PC intervention details | Outcome categories, measures and results | | |
| --- | --- | --- | --- | --- |
| **Controlled Intervention Studies (n=11)** | | | | |
| Coderch et al (2016)^20^ | Integrated, proactive program of care for complex chronic patients, based on four strategies:  - Patient identification: distribution of complex chronic patients lists assigned to primary care providers with a label in the EHR (1)  - Proactive actions in PC: individualized care plan in unique electronic medical record for providers (2)  - Access and coordination between different levels of care: outpatient day hospital unit for direct access of patients from PC and emergencies, support systems for discharges from hospitals and emergencies (3)  - Organizing culture: workshops and shared communication channels (4)  Control group: no strategy applied; partial intervention: strategies 1 and 3; full intervention: strategies 1, 2 and 3. (Assumption: all groups under strategy 4) | HC costs & util. | [1^st^ year, all complex chronic patients, control (CG) vs partial intervention (PI) vs full intervention (FI), (p < 0.05 significance against control group/ or between partial and full intervention noted]  [average per person]  - Primary care visits (non-urgent): 17.90 vs 18.40 (p<0.05 diff PI vs FI) vs 19.96 (p<0.05 diff vs CG)  - Primary care (urgent visit): 1.30 vs 1.33 vs 1.35  - Number of prescriptions: 91.19 vs 90.28 vs 92.16  - Total expenditure prescriptions: 1464.46 vs 1454.19 vs 1431.05  [average per 100 people]  - Hospital urgent visit: 79.76 vs 77.12 vs 83.84  - Urgent readmission <72 hrs.: 5.59 vs 5.10 vs 6.73  - Acute hospital admission: 23.07 vs 18.06 (p<0.05 diff vs CG) vs 22.32 (p<0.05 diff PI vs FI)  - Acute hospital stay: 178.91 vs 114.52 (p<0.05 diff vs CG) vs 159.39 (p<0.05 diff PI vs FI)  - Hospital readmissions <30 days: 2.90 vs 1.91 vs 2.69  - % deaths: 8.07 vs 8.14 vs 8.79 | [2^nd^ year, all complex chronic patients, control (CG) vs partial intervention (PI) vs full intervention (FI), (p < 0.05 significance against control group/ or between partial and full intervention noted]  [average per person]  - Primary care visits (non-urgent): 15.28 vs 16.04 (p<0.05 diff PI vs FI) vs 17.44 (p<0.05 diff vs CG)  - Primary care (urgent visit): 1.41 vs 1.28 vs 1.27  - Number of prescriptions: 85.06 vs 86.89 vs 88.65 (p<0.05 diff vs CG)  - Total expenditure prescriptions: 1412.46 vs 1478.05 vs 1467.50  [average per 100 people]  - Hospital urgent: 80.61 vs 80.61 vs 80.02  - Urgent readmission <72 hrs.: 6.32 vs 6.70 vs 5.08  - Acute hospital admission: 19.76 vs 18.76 vs 19.61  - Acute hospital stay: 139.56 vs 128.91 vs 147.96  - Hospital readmissions <30 days: 2.70 vs 1.76 (p<0.05 diff PI vs FI) vs 3.15 (p<0.05 diff PI vs FI)  - % deaths: 7.74 vs 7.83 vs 7.16 |
| Prestes et al (2017)^24^ | - Diabetes training course for physicians: Physicians in the IG attended an online course with 14 compulsory and 12 optional modules, plus 8 h of practical activities completed at a national reference centre  - Nurse education: Nurses in the IG attended in person a 5-day full-time intensive theoretical and practical course  - Patient follow-up and call centre activities: Participants in the IG were seen every three months in a programmed visit; to decrease dropouts, the call centre called them by telephone to remind them of their next appointment. Once a year, the call centre gave each patient an appointment for cardiovascular and ophthalmological controls done at the hospital at the same morning  - Data monitoring: Qualidiab data system was used to verify the impact of the diabetes education intervention and the data collected is also useful to allocate resources (human and financial) considering real demand | Pop. health | [Mean values for IG, basal vs 12-months (p value), statistically significant only]  - Diastolic BP (mmHg), 80.7 vs 77.8 (p = 0.006)  - Glycemia (mg/dL): 161 vs 143.1 (p = 0.02)  - HbA1c (%): 7.65 vs 7.18 (p = 0.004)  - Total cholesterol (mg/dL): 196.9 vs 182 (p = 0.001)  - LDL-c (mg/dL): 117 vs 107.8 (p = 0.05)  - Triglyceride (mg/dL): 201.2 vs 175.5 (p = 0.05)  Non statistical differences for Systolic BP, Creatinine, Proteinuria, and HDL-c | [% of patients who achieved treatment goals, control vs intervention at 12 months, statistically significant only]  -SBP < 130/80 mmHg: 59% vs 73% (p = 0.04)  - HbA1c < 7%, 36% vs 57% (p = 0.004)  Non statistical differences for DBP<80 mmHg, glycaemia < 100mg/dL, Cholesterol <200mg/dL, Triglyceride <150mg/dL |
|  |  | HC costs & util. | [% of patients treated, control vs intervention at 12 months, statistically significant only]  - Dyslipidemia, treated: 48% vs 74% (p = 0.01)  - Eye test, 75.3% vs 100% (p=0.000)  - CV evaluation, 68.9% vs 98.1% (p=0.000)  Non-statistically significant differences for dyslipidemia treated under target or any hypertension treatments. | |
| Ruescas-Escolano et al (2014)^25^ | Organizational intervention (based on the Chronic Care Model, Stanford Expert Patient Programme and Kaiser Permanente Model):  - Team-based work, active and informed patient, clinical guideline-based recommendations, shared decision making with the patient, use of EMR which allows for following control indicators and risk stratification  Professional training innovation:  - 4 workshops (every 3 months), monthly clinical update sessions, online tutorials led by a cardiologist, relevant documents and protocols available through the intranet | Pop. health | [Adjusted relative risk, 95% CI (p-value), intervention group]  - Smoking: 15.7, 4.20-58.70 (p<0.001)  - Low-density-lipoprotein cholesterol: 2.98, 1.48-6.02 (p=0.002)  - Systolic BP: 1.97, 1.21-3.23 (p=0.007)  - Diastolic BP: 1.51, 0.65-3.50 (p=0.342) | |
| **Observational Cohort or Cross-Sectional Studies (n=16)** | | | | |
| Goff et al (2017)^34^ | - The Buena Salud enhanced primary care team consisted of 2 registered nurse care managers, 2 medical assistants trained as community health workers, and a caseworker, and:  - Offered patients education and coaching for disease self-management  - Provided additional contact with patients in the clinic and at home  - Interfaced with diabetes specialists for decision support  - Used electronic health registries to identify patients in need of care and services  - Utilized linkage to community-based support services | Pop. health | [Difference in differences (95% CI), p-value]  - Change in mean diastolic BP: 2.5 (0.8 to 4.3), p = .004  No statistically significant changes for A1C measures, lipid measures, or other blood pressure measures | |
|  |  | HC costs & util. | [Difference in differences (95% CI), p-value]  - Adjusted microalbumin/creatinine ratio ever within 12 months (yes/no): 22.2% (6.7 to 37.8), p=.008  No statistically significant changes for A1C tests, lipid panels, ED visits, or hospitalizations | |
| Maeng et al (2013)^38^ | **Patient-centered primary care:**  – Provider-led, team-delivered care  – Patient and family engagement  – Enhanced access and scope of services  – HIT optimized preventive and chronic care  **Population management:**  – Population segmentation and risk stratification  – Case management for complex, comorbid conditions  – Disease management  – Preventive care  **Medical neighborhood:**  – Micro-delivery referral systems  – Complete care (360 degrees) delivery systems including nursing homes, emergency departments, hospitals, home health, and pharmacies, among others  **Quality outcomes:**  – Patient satisfaction  – HEDOS and bundled chronic disease metrics  – Preventive care metrics  **Value-based reimbursement model:**  – Fee for service with P4P payments for quality outcomes  – Physician and practice transformation stipends  – Quality-based gainsharing | Patnt. satisf.. | [Adjusted differences in % of responses, PHN vs Non-PHN, significant at P<0.05]  Perceived changes in care delivery:  – Noticed difference in care, 13.8%  – Noticed difference in care coordination, 19.6%  – Noticed difference in service, 16.8%  – Feel quality is higher than before, 10.4%.  Usual source of care:  – Doctor's office, 15.4%;  – ER, -12.3%.  Non-statistically significant differences for Access to care or PCP performance | |
| Maeng et al (2012a)^39^ |  | Pop. health | [Odds ratios (95% CI), for the risk of patients to suffer one of these events, associated to the conversion into a PHN, * = significant at 1% level]  – Amputation, 0.178* (0.048-0.664)  – End-stage renal disease, 0.688* (0.518-0.915)  – Myocardial infarction, 1.067 (0.993-1.146)  – Stroke, 0.986 (0.946-1.027) | |
| Phillips et al (2014)^42^ | Implementation of the Illinois Medicaid Health Connect (IHC, a primary care case management program for Medicaid that offered enhanced fee-for-service, capitation payments, performance incentives, and practice support) and Your Healthcare Plus (YHP, a complementary disease management program) programs. Nearly all YHP members were also in IHC.  – Every beneficiary required to select a primary care clinician  – Monthly care management fees: $2 for children, $3 for adults, $4 for disabled or elderly beneficiaries  – Pediatric claims paid within 30 days; adult claims paid within 60 days  – Quarterly academic detailing about the administration of the program and clinical care of the patient population for primary care clinicians  – Multiple online tools such as registries and report cards to assist clinicians with population-based management  – IHC providers required to make preventive care available within 5 weeks of request (or 2 weeks for infants younger than 6 months), urgent care appointments within 24 hours, appointments for non-serious complaints within 3 weeks, and follow-up visits within 7 days of discharge from an emergency department or hospital  – Practices required to provide and coordinate maternal and child health services  – Practices required to institute an action plan for enrollees with chronic diseases  – Quality-based bonus payments | HC costs & util. | – Estimated gross savings, 2007-2010: IHC, $237 million; YHP, $518 million; Medicaid overall, $1.46 billion  – Rate estimated annual savings, % increase, 2007 vs 2010: IHC, 2.5% vs 6.5%; YHP, 3.5% vs 8.6%.  [% change 2006-2010, no mention of significance]  – Adjusted hospitalization rate: IHC-eligible beneficiaries, -18.1%; YHP-beneficiaries, -9.7%  – Bed-day rate: IHC-eligible beneficiaries, -15.6%; YHP-beneficiaries, -13.4%.  – Avoidable hospitalizations: YHP-beneficiaries, -16.8%  – Adjusted ED visit rate: IHC-eligible beneficiaries, -5.0%; YHP-beneficiaries, -4.6%  [Quality measure changes for Illinois Health Connect patients, 2007-2010, % of change of patients appropriately treated, authors mention "significant" changes for all measures]  – Diabetes nephropathy screen, 8.9%  – Diabetes annual HbA1c, 23.4%  – Diabetes retinal eye exam, 51.7%  – Ischemic vessel disease with annual lipid profile, 34.7%  – Mammogram, 87.4%  – B-blocker for post-acute myocardial infarction, -13.7  – Asthma control medication, 6.2%  – Cervical cancer screening, 65.7%  – Colonoscopy, 77.2%. | |
| Wensing et al (2017)^43^ | Implementation of the GP-centered care (GPCC) program, which included the following elements:  – **Engaged leadership**: GPCC is arranged in special contracts, which have been developed by organisations of GPs in collaboration with health insurers  – **Data-driven improvement**: physician participates in quality circles: small groups of physicians who receive feedback on their prescribing, evidence based information and plan improvements. The practice has a data-orientated quality system and decision support for prescribing medication  – **Empanelment**: physician participates in disease management programs (DMP) which concern panels of patients with diabetes, asthma/COPD, and coronary heart disease; participation by patients is voluntary and based on written informed consent. After consent, patients are added to the panel of patients in GP-centred care  – **Team-based care**: Disease management programs imply enhanced participation of practice assistants in clinical work, who are encouraged to take part in an additional training program (VERAH) for better management of patients with chronic diseases. Practices who have their assistants qualified are entitled to receive a financial bonus  – **Patient-team partnership**: Self-management support, where patients are offered a validated educational program, which comprises informative group meetings  – **Population management**: Feedback and benchmarking on prescribing is supported by short written evidence reports. Recommendations are strictly evidence-based and not influenced by industry. Prompts in the software of a practice support use of generic and discounted drugs where eligible  – **Continuity of care**: Referrals to medical specialists are preceded by relevant diagnostic procedures and treatments and, in case of referral, the findings are clearly communicated to medical specialists and backwards  **– Prompt access to care**: practice organization has a number of clinical facilities (e.g., spirometer), daily consultation hours, up-to-date information technology. Patients benefit from shorter waiting times and absence of out-of-pocket payments for medication  – **Comprehensiveness and care coordination**: physician is trained in primary care-relevant domains (e.g., pain treatment, communication skills) and participates in continuing education  – **Template of future**: Participation in GPCC is a voluntary choice of physicians and patients. For the Family Practice, is associated with about 40% increased reimbursement for enrolled patients as lump sum payment without pre-specified maximum. | HC costs & util. | [Impact of the program at T2 (year 5 of implementation), adjusted difference (SE) [95% CI], intervention vs control, statistically significant only at p<0.05/X)]  – Mean costs of medication therapy in ambulatory care in observed year (euro), -85.39 (0.009) [-0.064; -0.028]  – Mean total costs of hospital admission in year (euro), −44.30 (0.003) [−0.017; −0.002]  [Impact of the program at T2 (year 5 of implementation), adjusted difference (SE) [95% CI], intervention vs control, statistically significant only at p<0.05/X)]  – Mean number of visits to the FP, +1.98 (0.163) [1.659;2.297]  – Mean number of prescribed drugs, +0.071 (0.022) [0.028; 0.116]  – Mean percentage of prescriptions that should be avoided per FP, -0.699 (0.041) [-0.779; -0.619]  – Mean number of contacts with medical specialists with referral from FP, -0.455 (0.013) [-0.480; -0.428]  – Mean number of contacts with medical specialists without referral, -1.528 (0.076) [-1.667; -1.378]  – Mean number of hospital admissions, −0.017 (0.006) [−0.085; −0.061]  – Mean percentage of avoidable hospital admissions of all admissions, −1.165 (0.145) [−0.880; −1.449]  – Mean number of days in hospital, −0.438 (0.083) [−0.276; −0.599]  – Mean number of hospital admissions within 4 weeks after a previous hospital admission, −0.007 (0.012) [−0.070; −0.020] | |
| Dale et al (2016)^31^ | \| Comprehensive Primary Care Initiative’s change package specifies four primary drivers of change:  - The five comprehensive primary care functions (i.e., access and continuity, planned care for chronic conditions and preventive care, risk-stratified care management, patient and caregiver engagement, and coordination of care across the medical neighborhood)  - Enhanced accountable payment, including a non-visit based per beneficiary per month payment and the opportunity to share in any savings  - Continuous improvement driven by data  - Optimal use of health IT. \| \| --- \| \| | HC costs & util. | [Adjusted difference-in-differences (95% CI), relative difference, P Value]  **Total Medicare expenditures ($/beneficiary/mo)**  - Without initiative care-management fees: -11 (-23 to 1), -1%, p=0.07  - With initiative care-management fees: 7 (-5 to 9), 1, p=0.27  **Utilization (annualized rate/1000 beneficiaries)**  - Hospitalizations: -5 (-12 to 2), -2, p=0.13  - Outpatient emergency department visits: -4 (-13 to 5), -1, p=0.40  - Primary care visits in all settings: -230 (-357 to -103), -3, p<0.001  - Specialist visits in all settings: -6 (-152 to 141), 0, p=0.94  - Admissions for ambulatory-care-sensitive  conditions: 1 (-2 to 3), 1, p=0.54  - % Likelihood of 30-day readmission after discharge: 0 (-0.9 to 0.3), -2, p=0.30  - % Likelihood of 14-day follow-up visit with any provider after discharge: 0 (-1.5 to 0.6), -1, 0.40  **Quality of care**  - None of the test performed (diabetes patients): -0.6 (-1.2 to 0.1), -11, p=0.03  Non-significant differences for tests of HbA1c, lipid levels, eye exam, urine protein or all tests performed (for diabetes patients), and for lipid levels on ischemic vascular disease patients  Continuity of care  - No significant difference for PC visits at attributed practice  [Difference-in-differences, percentage points, P Value]  - Timely appointments, care, and information: 2.1, p=0.05  - Support for patients in caring for their own health: 3.8, p<0.001 - Discussion of medication with patients: 3.2, p=0.006  Non significant differences for providers’ communication with patients, providers’ knowledge of care received from other providers and patient rating of providers | |
| **Case Control Studies (n=1)** | | | | |
| Freytag et al (2016)^44^ | The GP-centered healthcare program complements regular GP care (“add-on contract”). The elements of the program for participating GPs were:  – Mandatory participation in clinical GP–peer group trainings (quality circles; 3 per year, 2h each session, provided by a professional trainer)  – Obligatory use of a specific IT–pharmacotherapy tool to support rational pharmacotherapy  – Bonuses to support the prescription of generics and recommended substances and to limit growth in the overall number of prescriptions  – Financial incentives to employ trained health-care assistants in patient-care (‘VERAH’, a qualification consisting of 3 years of vocational (on-the-job) training including half a day of school per week and about 200 units of additional training in patient-care)  – A lump sum payment for each patient enrolled in the program (€2/three months)  – Additional lump sum payments stratified according to the individual degree of morbidity of the enrolled patients (€6, €3, and/or €2, per three-month period). The financial incentives were offered in addition to the regular payment system for GP care. The participating patients committed themselves to choose the contracted GP as a first-line contact to the health care service without being bound to the program by any financial or other incentives. | HC costs & util. | Costs  [Comparative outcomes DiD for healthcare costs, mean values per patient, Absolute intervention effect (p-value), statistically significant only, unless noted]  – Mean total direct costs per patient, +€83 (p=0.215, non-significant)  – Cost of GP consultations, +€27 (p<0.001)  – Specialist consultations, +€22 (p=0.0385)  – Cost of drug prescriptions, - €44 (p=0.001)  Utilization  [Secondary comparative outcomes DiD for resource utilization, mean values per patient, Absolute intervention effect (p-value), statistically significant only]  – GP consultations, 3.7 (p<0.001)  – Specialist consultations, 0.5 (p<0.001)  – Hospital care, 0.03 (p=0.006);  – Remedies, 0.03 (p=0.034).  [Secondary comparative outcomes DiD for care coordination, Absolute intervention effect (p-value), statistically significant only]  – Share of patients consulting more than one GP, OR 0.59 (p<0.001)  – Share of specialist consultations w/o referral, Mean Diff -0.01 (p<0.001)  – Number of home visits by GPs per patient, MD 0.08 (p<0.001)  – Number of DMP participants, MD 0.07 (p<0.001)  [Secondary comparative outcomes DiD for pharmacotherapy, Absolute intervention effect (p-value), statistically significant only]  – Number of different medications for patients, 0.06 (p=0.035)  – Share of patients with 5 or more different medications, 1.15 (p<0.001) | |
| **Before and After (Pre-Post) Studies with no control (n=9)** | | | | |
| Conrad et al (2008)^47^ | Access initiative included the following reforms:  - Advanced access to primary care (appointments with a patient’s PCP at the preferred time of the patient)  - Direct access to most specialties without primary care gatekeeping;  - Changes in physician compensation  - Primary care system redesign to control costs  - Patient-provider secure messaging through the MyGroupHealth enrollee website including physician financial incentives for secure messaging patients - Internet access for enrollees to their electronic medical records (EMRs) through MyGroupHealth  - Health promotion information on the MyGroupHealth secure website  Three of the components directly addressed physician productivity:  - Primary care redesign, to reposition the group practice to be market competitive by restructuringt primary care  - Changes in physician compensation, from 100% guaranteed salary to 80% guaranteed salary plus additional variable compensation up to 120% of the guaranteed base determined by number and intensity of patient encounters  - Patient-physician secure messaging thorugh MyGroupHealth | HC costs & util. | [Regression coefficients (SE), p-value, Post-pre difference]  - Panel size per FTE: 302.20 (48.99), p<.001  - Work relative value unit (RVU) per visit: 0.24 (0.02), p<.001  - Visit per FTE: -69.53 (17.30), p<.001  - RVU per FTE: 70.32 (38.75), non-significant  - Per member, per quarter cost: -66.01 (6.70), p<.001 | |
| Engel et al (2016)^48^ | Geriatrics in Primary Care complements the PACT medical home interdisciplinary model by:  - Adding an on-site geriatric physician (0.3 full-time employee equivalent (FTEE)) and geriatric RN care manager (1.0 FTEE) to existing teams comprising primary care providers, nurses, clinical pharmacists, mental health professionals, social workers, and nutritionists.  - Emphasizing informed use of existing resources, simplification and defragmentation of care plans, personal relationships with professional colleagues, ready availability of clinical team members to veterans, care continuity, and better planning for care transitions.  - Providing comprehensive consultation and care management undertaken jointly with the primary care team  - Providing focused evaluations for individuals with memory concerns  - Proactive telephone contact with veterans and caregivers, ready access to primary care colleagues, and informed use of telephone follow-up intended to enhance care while reducing nonessential clinic visits | HC costs & util. | [Mean number of visits ± standard deviation (P-value)]  Before enrolment:  - Physician specialty clinics: 7.6 ± 9.8  - Primary Care Physician or Nurse Practitioner: 3.1 ± 1.5  Postenrollment Year 1  - Physician specialty clinics: 4.7 ± 5.0 (.01)  - Primary Care Physician or Nurse Practitioner: 3.3 ± 1.5 (.50)  Postenrollment Year 2  - Physician specialty clinics: 4.5 ± 6.1 (.03)  - Primary Care Physician or Nurse Practitioner: 2.9 ± 1.5 (.06) | |
| Maeng et al (2012b)^52^*  *Same intervention as Maeng 2013 and 2012a above | **Patient-centered primary care:**  – Provider-led, team-delivered care  – Patient and family engagement  – Enhanced access and scope of services  – HIT optimized preventive and chronic care  **Population management:**  – Population segmentation and risk stratification  – Case management for complex, comorbid conditions  – Disease management  – Preventive care  **Medical neighborhood:**  – Micro-delivery referral systems  – Complete care (360 degrees) delivery systems including nursing homes, emergency departments, hospitals, home health, and pharmacies, among others  **Quality outcomes:**  – Patient satisfaction  – HEDOS and bundled chronic disease metrics  – Preventive care metrics  **Value-based reimbursement model:**  – Fee for service with P4P payments for quality outcomes  – Physician and practice transformation stipends  – Quality-based gainsharing | HC costs & util. | [Total, per-member, per-month allowed cost, Coefficient ($) (95% CI), Without Rx coverage interaction, significant only]  – PHN exposure: 1-6 mo, -24.08 (-46.95 to -1.22) (P<0.1)  – PHN exposure: >24 mo, -59.70 (-126.50 to 7.09) (P<0.1)  – Rx coverage: 176.83 (153.04-200.62) (P<0.05)  [Total, per-member, per-month allowed cost, Coefficient ($) (95% CI), With Rx coverage interaction, significant only]  – PHN Exposure: 1-6 mo, -38.11 (-76.62to -0.40) (P<0.1)  – PHN exposure: 7-12 mo, -37.13 (-81.08 to 6.81) (P<0.1)  – PHN exposure: >24 mo, -108.3 (-183.32 to -33.28) (P<0.01)  – Rx coverage, 164.75 (139.25-190.26) (P<0.01)  – Rx coverage X PHN exposure: 13-24 mo, 37.13 (-1.56 to 75.83) (P<0.1)  – Rx coverage X PHN exposure: >24 mo, 63.35 (18.78-107.91) (P<0.05) | [Estimated Savings, % difference (95% CI), Without Rx coverage interaction, significant only]  – PHN Exposure: 13-24 mo, 4.3 (-0.1 to 8.6) (P<0.1)  – PHN Exposure: >24 mo, 6.7 (1.2-21.1) (P<0.05)  – Overall, 4.3 (0.4-8.3) (P<0.05)  [Estimated Savings, % difference (95% CI), With Rx coverage interaction, significant only]  – PHN Exposure: 13-24 mo, 7.1 (2-12.3) (P<0.01)  – PHN Exposure: >24 mo, 10.8 (4.7-17) (P<0.01)  – Overall, 7.1 (2.6-11.6) (P<0.01) |
| Ralston et al (2009)^53^ | Implementation of the Access Initiative, which included the following changes in service delivery:  – **Web Access for patients**: Secure e-mail with physicians; Medical record access; Medication refills; Appointment scheduling; Discussion groups; Health promotion information  – **Advanced Access**: Appointments with a patient’s primary care physician at the preferred time of the patient  – **Primary Care Redesign**: Reduce variation in physician productivity; Team members to work at high end of expertise; Reduce wait times for patients on the phone and during appointments; Increase physician influence and accountability for daily practice environment  – **Direct Access**: Direct Access to specialty care (16 specialties), or removal of gate-keeping  – **Physician Payment Reform**: 80% to 120% variable compensation around baseline salary; Variance dependent on patient satisfaction, physician productivity, and coding accuracy | HC costs & util. | [% "Not a problem" response (p-value), "Getting Needed Care" section, rollout vs post-initiative, statistically significant only]  – Get care you or your dr believed necessary, 77.8 vs 81.5 (p <0.01)  – Get care while awaiting approval from health plan, 53.8 vs 62.9 (p<0.01),  – Section Composite score (adjusted), 67.0 vs 69.7 (p<0.01).  [% "Always/Usually" response (p-value), "Getting Care Quickly" section, rollout vs post-initiative, statistically significant only]  – Get appointment for routine care as soon as you wanted, 80.0 vs 81.8 (p=0.04)  – Get taken to exam room within 15 min of appt time, 44.2 vs 72.7 (p<0.01);  – Section Composite score (adjusted), 73.8 vs 81.8 (p<0.01) | |
|  |  | Patnt. satisf. | [% "Very Satisfied" response, rollout vs post-initiative, statistically significant only]  – Able to see personal doctor when needed care, 66.6 vs 71.3 (p<.01)  – Time spent on phone scheduling appointment, 62.7 vs 70.3 (p<.01)  – Time to appointment, serious problem, 68.7 vs 71.7 (p<.01)  – Time to appointment, minor illness, 65.1 vs 68.6 (p<.01)  – Time to appointment, routine exam, 57.1 vs 62.5b (p<.01)  – Ease of getting necessary care, 64.6 vs 69.5 (p<.01)  – Rate all your health care, 69.1 vs 72.2 (p<.01)  – Rate your health plan, 59.1 vs 61.3 (p=.03)  – Overall opinion of Group Health, 60.8 vs 64.2 (p<.01) | |
|  |  | Prov. satisf. | [Mean score (out of 5 points) (SD) (p-value), Rollout vs Post-initiative , statistically significant results only]  – I am confident that Group Health leaders are taking Group Health in the right direction, 2.80 (1.06) vs 3.38 (0.97) (p <.01)  – Group Health’s efforts to improve the quality of service are resulting in improvements, 3.28 (1.03) vs 3.70 (0.93) (p<.01);  – I am proud of the quality of service we provide to our patients and other customers, 3.65 (1.03) vs 3.90 (0.89) (p <.01);  – I would recommend Group Health to my friends as a good place to work/practice, 3.22 (1.19) vs 3.70 (1.09) (p=.05) | |

Notes: Pop. Health = Population health outcomes; HC costs & util. = Healthcare costs and utilization outcomes; Patnt. satisf. = Patient satisfaction outcomes; Prov. satisf.= Provider satisfaction outcomes; CG = Control group; IG = Intervention group; EHR = Electronic health record; ER = Emergency room; PHN = PatientHealthNavigator; D/SBP = Diastolic/Systolic blood pressure; HbA1C = glycated hemoglobin; HIT = Health information technologies;
